# Spectrum of Fates: a new approach to the study of the developing zebrafish retina

**DOI:** 10.1242/dev.104760

**Published:** 2014-05

**Authors:** Alexandra D. Almeida, Henrik Boije, Renee W. Chow, Jie He, Jonathan Tham, Sachihiro C. Suzuki, William A. Harris

**Affiliations:** 1 Department of Physiology, Development and Neuroscience, University of Cambridge, Cambridge CB2 3DY, UK; 2 Institute of Neuroscience, Chinese Academy of Sciences, Shanghai 20031, China; 3 Department of Biological Structure, University of Washington, Seattle, WA 98195-7420, USA

**Keywords:** Cell fate, Differentiation, Fluorescent proteins, Neuron, Retina, Zebrafish

## Abstract

The ability to image cells live and *in situ* as they proliferate and differentiate has proved to be an invaluable asset to biologists investigating developmental processes. Here, we describe a Spectrum of Fates approach that allows the identification of all the major neuronal subtypes in the zebrafish retina simultaneously. Spectrum of Fates is based on the combinatorial expression of differently coloured fluorescent proteins driven by the promoters of transcription factors that are expressed in overlapping subsets of retinal neurons. Here, we show how a Spectrum of Fates approach can be used to assess various aspects of neural development, such as developmental waves of differentiation, neuropil development, lineage tracing and hierarchies of fates in the developing zebrafish retina.

## INTRODUCTION

The dynamic, four-dimensional imaging of developmental processes *in vivo* has given us unprecedented views into the underlying mechanisms that drive the formation of complex tissues such as the brain. Advances in highly sensitive fluorescence microscopy when combined with the new generations of differently coloured fluorescent proteins (Giepmans et al., 2006) mean that we can track several proteins simultaneously through time. Brainbow technology, which is based on combinatorial expression of fluorescent proteins (FPs), allows us to trace hundreds of different cells and their processes in the brain by virtue of their unique spectrum of colours (Livet et al., 2007). New techniques such as ScaleA2, SeeDB and Clarity, enable high resolution imaging of entire adult mammalian brains in 3D by rendering intact brain tissue optically transparent (Hama et al., 2011; Chung et al., 2013; Ke et al., 2013), but can only capture snapshots of fixed tissue. Recently Zebrabow, which combines Brainbow technology with the optical transparency of live zebrafish embryos, is enabling developmental biologists to image and distinguish all cells in real time *in vivo* by virtue of various colours (Pan et al., 2013). However, as Brainbow is based on a technology in which stochastic combinations of FPs are expressed in cells, the colour of the cell does not carry any particular significance.

Here, we describe a Spectrum of Fates approach to study zebrafish retinal development. In the two Spectrum of Fate lines of fish that we created (SoFa1 and SoFa2), a distinct fluorescent colour is associated with each of the major cell types. This is achieved by driving the expression of differently tuned FPs from the promoters of a set of transcription factors (TFs), each of which is expressed in one or more cell types. Thus, in SoFa lines, we can simultaneously identify all cells, but now each type is specified by a defined spectrum. Spectrum of Fates is useful to study the global dynamics of development and particularly the interactions between cell types. This paper shows how such an approach can be used to shed new light on the acquisition of neuronal fates and the early steps of cell differentiation.

## RESULTS

### The transgenes

The vertebrate retina comprises five major neuronal types, photoreceptors (PRs), horizontal cells (HCs), bipolar cells (BCs), amacrine cells (ACs) and retinal ganglion cells (RGCs), and a single type of glial cell, the Müller glia. In the past two decades, through a combination of functional analysis, genetic screens and bioinformatics approaches, numerous TFs necessary to specify retinal cell types have been identified, characterized and used in 4D live-imaging experiments to help us understand particular aspects of cell fate acquisition and early differentiation. To generate a SoFa retina, we chose three genes: *Atoh7*, *Ptf1a* and *Crx*. Atoh7 is a bHLH transcription factor required for the genesis of RGCs (Kanekar et al., 1997; Brown et al., 2001; Kay et al., 2001; Wang et al., 2001; Poggi et al., 2005). Ptf1a, which is also a bHLH transcription factor, is necessary and sufficient for the generation of retinal inhibitory neurons, the ACs and HCs (Fujitani et al., 2006; Dullin et al., 2007; Nakhai et al., 2007; Jusuf et al., 2011). Crx is a homeodomain transcription factor with multiple functions during retinal development, which is expressed in BCs and PRs in zebrafish (Liu et al., 2001; Shen and Raymond, 2004). We reasoned that by using a combination of *Atoh7*, *Ptf1a* and *Crx* promoter sequences each driving a different FP, we could label all of the main retinal neurons in distinct spectral combinations of colours.

**Fig. 1.**
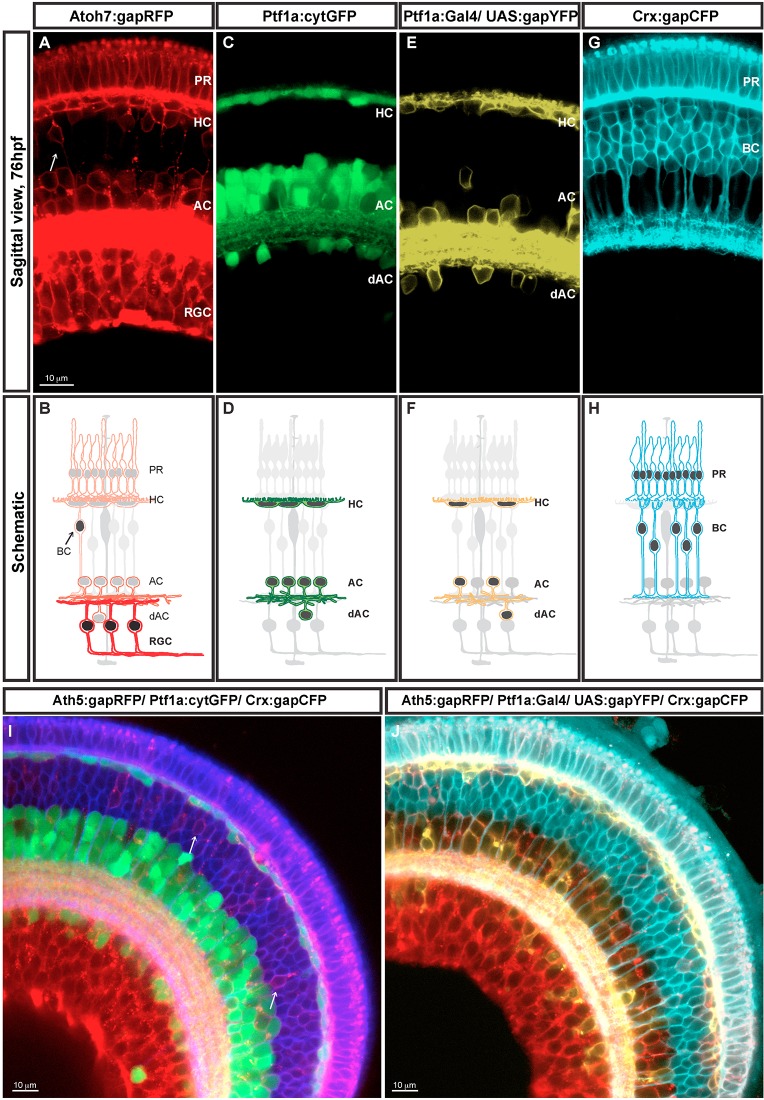
**SoFa transgenic lines.** Retina sagittal sections (76 hpf). Reporter protein expression profile and corresponding diagram of each of the four transgenes used to generate the SoFa lines (A-H). (A,B) gapRFP expression of Atoh7:gapRFP retinas. Arrow indicates Atoh7+ BCs. (C,D) cytGFP expression of Ptf1a:cytGFP retinas. (E,F) Variegated gapYFP expression of Ptf1a:Gal4/UAS:gapYFP retinas. (G,H) gapCFP expression of Crx:gapCFP retinas. SoFa fish were generated combining these individual transgenic lines. (I) In Sofa1 fish, which were generated by mating Atoh7:gapRFP, Ptf1a:cytGFP and Crx:gapCFP, retinal cells are labelled with a combination of membrane-tagged and cytoplasmic FPs. (H) In Sofa2 fish, which were generated by mating Atoh7:gapRFP, Ptf1a:Gal4/UAS:gapYFP and Crx:gapCFP, all retinal cells are labelled with a membrane-tagged FP. PR, photoreceptors; HC, horizontal cells; BC, bipolar cells; AC, amacrine cells; dAC, displaced amacrine cells; RGC, retinal ganglion cells.

In the previously described Atoh7:gapRFP transgenic line (Zolessi et al., 2006), the Atoh7 promoter drives the expression of membrane-tagged monomeric RFP in retinal progenitors prior to their last division. Invariably, the daughter cell with higher levels of gapRFP becomes a RGC (Fig. 1A,B, stronger RFP signal and stronger red in the schematic). This newborn RGC continues to upregulate gapRFP expression, whereas the other daughter often divides again, typically giving rise to PRs, HCs or ACs [RFP signal in AC, HC and PR (Fig. 1A), light red in the schematic (Fig. 1B)] and much less frequently to BCs (Fig. 1A,B, labelled with an arrow). However, although the *atoh7* mRNA is expressed only transiently during RGC genesis (Masai et al., 2000), the gapRFP protein is stable and therefore it is retained for a few days in all the cells that come from Atoh7-expressing progenitors, which includes RGC, AC, HC and PR cells, as shown in Fig. 1A,B.

Ptf1a is a transcription factor that is crucial for pancreas development, but is also strongly expressed in the retina (Lin et al., 2004). The Ptf1a:cytGFP and Ptf1a:Gal4/UAS:gapYFP transgenic lines were generated by BAC recombineering, in which the *ptf1a* regulatory regions drive the expression of cytGFP or Gal4-VP16 driving UAS:gapYFP (Godinho et al., 2005). These lines express green or yellow FP in young ACs and HCs soon after their final cell division (Godinho et al., 2005) (Fig. 1C,D). Endogenous *ptf1a* mRNA is expressed transiently, but the cytGFP or gapYFP fluorescent signal is retained by Ptf1a-expressing neurons for the first few days of development. Although the cytoplasmic GFP is expressed in all ACs and HCs, the expression of the membrane-tagged gapYFP is often variegated in both ACs and HCs due to the susceptibility of the UAS promoter to transcriptional silencing and DNA methylation (Akitake et al., 2011).

A Crx-MCFP DNA construct was used to generate a Crx:gapCFP transgenic line, in which the Crx regulatory regions drive the expression of membrane-tagged CFP, a cyan FP (Suzuki et al., 2013). The onset of Crx expression occurs in proliferating retinal progenitors, as well as in PRs and BCs (Shen and Raymond, 2004; Bernardos et al., 2007). In the Crx:gapCFP transgenic line, the gapCFP expression also follows the pattern of endogenous *crx* mRNA, with a slight delay (Fig. 1G,H). Crx is expressed in both cone and rod PRs in adult fish, and consequently the gapCFP fluorescent signal in PRs persists through adulthood.

### The SoFa lines

By mating, screening and then inbreeding these transgenics, we generated two multi-transgenic lines in which all retinal neurons are labelled with particular combinations of FPs. The SoFa1 line (Atoh7:gapRFP/Ptf1a:cytGFP/Crx:gapCFP) shows a combination of membrane-tagged and cytoplasmic FPs (Fig. 1I). In SoFa1, all RGCs are labelled with gapRFP and therefore show a red membrane labelling; all inhibitory neurons are labelled by both gapRFP and cytGFP, and thus show a red membrane and a green cytoplasmic labelling; all BCs are labelled with gapCFP, so all show blue membrane labelling. A rare subset of BCs also expresses Atoh7; therefore, these BCs show a purple membrane labelling, corresponding to the combination of red and blue (Fig. 1I, arrows); finally, all PRs are co-labelled with both gapRFP and gapCFP, and consequently show a purple membrane labelling.

In SoFa2 fish (Atoh7:gapRFP/Ptf1a:Gal4/UAS:gapYFP/Crx:gapCFP) all retinal cells are labelled with membrane-tagged FPs (Fig. 1J). However, given that the expression of gapYFP is variegated, some inhibitory neurons have red and yellow membrane labelling, while others only have red membrane labelling. In this transgenic line, Crx:gapCFP-expressing cells are pseudo-coloured with cyan, and consequently all PRs show a brighter cyan/white colour, resulting from the combination of red and cyan. Though the variegation in Ptf1a:Gal4/UAS:gapYFP expression means that not all cells are labelled, this type of line is particularly useful if one wants to combine fate knowledge with dynamic events such as signalling, which could be fluorescently reported in the cytoplasm.

### A wave of retinal differentiation

**Fig. 2.**
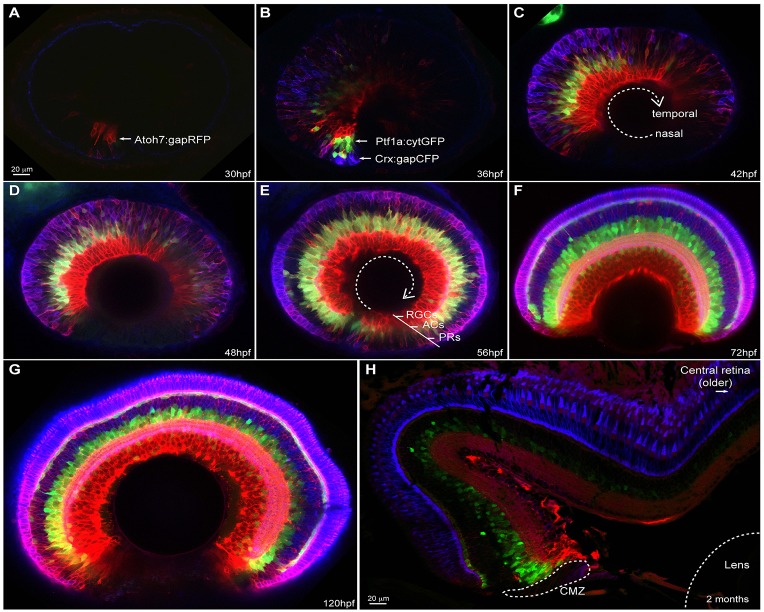
**SoFa1 spatial-temporal expression highlights the retina differentiation wave.** Sagittal sections of SoFa1-expressing retinas at various developmental stages. (A-E) Images showing the progression of the retinal ganglion cell (RGC), amacrine cell (AC) and photoreceptor (PR) differentiation wave from the nasal to the temporal retina. (F,G) From 72 hpf, the initial phase of retinal neurogenesis is complete and all five major cell types are easily identifiable by their colour and position in the retina. Throughout development, the retina keeps growing in size. (H) Newly differentiated multicoloured cells are generated only in the retina periphery, in the ciliary marginal zone (CMZ), as shown in the transverse section of a 2-month-old SoFa1 retina.

Previous work has shown that zebrafish retinal cells both leave the cell cycle and begin to differentiate in a wave-like manner that starts at the ventronasal centre of the retina and then spirals out ending in the ventrotemporal periphery (Hu and Easter, 1999; Schmitt and Dowling, 1999; Neumann and Nuesslein-Volhard, 2000). Although there is a relationship between cell birth and cell fate acquisition, these two events are distinct and may rely on largely independent mechanisms (Gomes et al., 2011; He et al., 2012). For example, one widely held view is that the cellular environment into which a retinal cell is born affects the probability that it will choose a particular fate. Up until now, however, it has not been possible to see all the cells differentiating simultaneously with respect to each other in live embryos. In the SoFa1 retina, gapRFP expression is first detected in the ventronasal retina adjacent to the choroid fissure at ∼28-30 hpf (Fig. 2A). While gapRFP expression begins to spread through the retina over the next few hours, expression of gapCFP, followed shortly after by the expression of cytGFP, also starts ventronasally and then follows the gapRFP expression around the retina until ∼72 hpf (Fig. 2B-F, supplementary material Movie 1 and Fig. S1). Only the ciliary marginal zone (CMZ), the neurogenic zone at the very periphery of the retina, remains undifferentiated. As the larvae grow, the fluorescence signals in the central retina of the two SoFa lines gradually disappear, with the exception of gapCFP in PRs (Shen and Raymond, 2004). However, in the retina periphery, where neurogenesis continues for as long as the fish grows, fluorescently labelled neurons of all types can still be visualized (Fig. 2H).

**Fig. 3.**
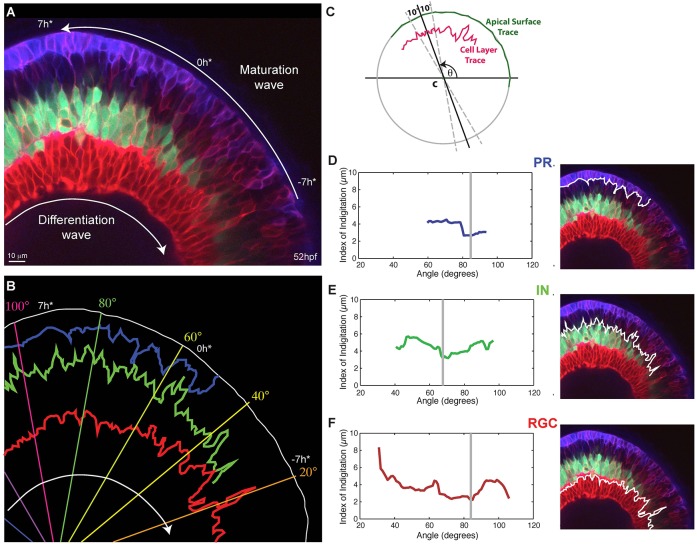
**Interdigitation of emerging cell types.** (A) Snapshot of a 52 hpf retina showing various degrees of interdigitation of the different cell types along the differentiation wave. The timescale, which references time in hours to the first appearance of the proto-IPL (0 h*), is based on matching the degree of lamination along the maturation wave to what we observe in our timelapse movies, where retinal lamination of a small section of the retina is followed over time (e.g. supplementary material Movie 2). (B) Traces of the apical surface (white), the basal side of the photoreceptor (PR) layer (blue), the apical side of the inhibitory neuron (IN) layer (green) and apical side of the retinal ganglion cell (RGC) layer (red) from when they first appear continuous. (C) A schematic showing how the Index of Interdigitation is calculated. (D-F) The Index of Interdigitation varies across the maturation wave for the basal side of the PR layer (D), the apical side of the IN layer (E) and the apical side of the RGC layer (F). The grey line marks the absolute minimum in each graph.

Four-dimensional movies of developing SoFa1 retinas demonstrate that although some cell types (e.g. RGCs) tend to differentiate before others (e.g. HCs and BCs), there is a substantial mixing in the generation of the different cell types (supplementary material Movies 1, 2). Indeed, frame-by-frame examination of such movies, suggests that there are regions of the retina where ACs and HCs begin to differentiate before PRs, while in neighbouring regions the reverse appears to be the case. These movies also show that at early stages of retinal differentiation, cells do not appear to migrate immediately into place. Instead, they adjust their position with respect to other differentiating cells, by as yet unknown mechanisms, to make more precise boundaries between cell layers. Maturation occurs in the wake of the differentiation wave, so in static images it appears to progress in the opposite direction (Fig. 3A). To begin to analyse this maturation process quantitatively, we devised an Index of Interdigitation, which is defined as the standard deviation of the maximal apical-basal position of the cell layer of interest over a finite segment of the retina, to measure the level of interdigitation of the different cell types across the developmental wave (Fig. 3B,C) and over time in a specified segment of the retina (supplementary material Movie 2). This analysis shows that each cell type goes through a period of relatively high interdigitation that then drops to a minimum as cellular layers coalesce (Fig. 3D-F). This minimum subsequently increases in the case of inhibitory cells, as HCs migrate apically to reach their final position, as well as in the case of RGCs, due to the migration of displaced ACs into the RGC layer. As similar trends in the way Index of Interdigitation can be seen across samples (data not shown), hallmarks in the curve of the Index of Interdigitation can be used to reference time in the developing retina independent of retinal position.

The finding that different cell types arise simultaneously in closely neighbouring areas of the retina, leading to the transient interdigitation of cell types before they sort into layers, matches the overall patterns of cell type histogenesis in the retinas of many species, with RGCs differentiating first, followed by ACs and then PRs (Holt et al., 1988; Belecky-Adams et al., 1996; Rapaport et al., 2004; He et al., 2012). What is striking, however, is that interdigitation is evident within relatively narrow segments of the wave. This is consistent with recent findings from the time-lapse analysis of clones in which different cell types are born simultaneously and occasionally even subvert the overall histogenetic order (Gomes et al., 2011; He et al., 2012). We propose that the clonal variability in histogenesis leads to initially high levels of interdigitation of different cell types. In any case, these results make it difficult to think of retinogenesis as a precisely regimented process in which cell types in any small region of the retina are born in perfect order and immediately migrate into their correct position.

### Live lineage tracing with cell fate identification

**Fig. 4.**
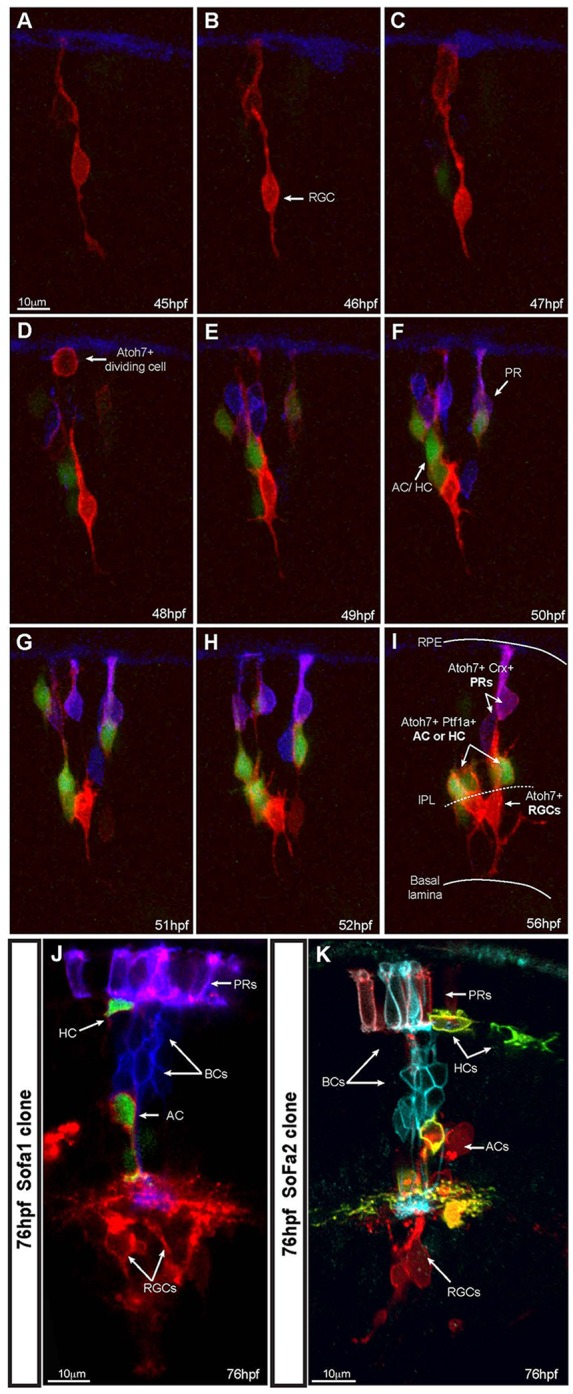
**SoFa lines allow precise identification of cell fate within developing clones.** (A-I) Time lapse series of a developing SoFa1 clone in an unlabelled wild-type host. We presume this clone arose from a single progenitor cell due to its final size. Imaging started at ∼45 hpf, corresponding to the time of the appearance of the first gapRFP-expressing cells (Atoh7+) and image stacks were collected every hour. Some Atoh7+ cells differentiate into RGCs, whereas others divide and start expressing gapCFP (Crx+) or cytGFP (Ptf1a+). At the onset of differentiation, it is possible to identify the final fate of these cells before they reach their final position: RGCs (red cells expressing increasing levels of gapRFP, arrow), inhibitory neurons (red and green cells), PRs (purple cells). Crx+ BCs did not yet arise through the duration of the movie: ∼12 h time-lapse movie; extended focus confocal image, *z* stack=25.5 μm; step size=1.5 μm. (J,K) Examples of SoFa clones at 76 hpf, when all cell types have differentiated and show their characteristic laminar position in the retina. (J) SoFa1: PRs (purple); BCs (blue); AC/dAC/HC (red and green); RGCs (red); single confocal image. (K) A SoFa2 clone: PRs (cyan and red); BCs (cyan); AC/dAC/HC (red and yellow); RGCs (red); 3D projection, *z* stack=40 μm, step size=2 μm.

Clonal analysis of retinal development has established a number of important principles, such as the pluripotency of retinal progenitors (Turner and Cepko, 1987; Holt et al., 1988; Wetts and Fraser, 1988). A detailed statistical analysis has more recently revealed that a simple intrinsic stochastic engine within equipotent retinal progenitors can provide an excellent fit to the size distributions of clones generated at various stages of retinal development (Gomes et al., 2011; He et al., 2012). However, because cell types have been much harder to assign definitively in such experiments, we are still some distance from generating a corresponding model that accurately predicts cell fate distributions in clones. Until we can unambiguously assign cell fate identity within clones, we will not be able to develop or test such a model. By transplanting blastomeres from SoFa lines to an unlabelled wild-type host, we can visualize multicolour clones and how they arise in the host embryo, as each newly generated daughter cell turns on the combinations of FPs that reveal its neuronal type unambiguously (except for some ACs and HCs, which express the same combination of colours but which can easily be distinguished by their position within the inner nuclear layer) (Fig. 4A-I, supplementary material Movies 3, 4). In Fig. 4 and in supplementary material Movie 3, it is also possible to see that there is no rigid order of fate determination, even within single clones, as there is a late, as well as an early, arising RGC, and a PR that differentiates at the same time as an AC. In this clone, we did not track cell division, but the addition of another label, such as H2B-GFP that labels all the cells in the clone, should allow us to test how birth order correlates with the relative differentiation of the cells that arise from terminal divisions within clonal trees. SoFa lines, we suggest, are excellent model systems with which to carry out such analyses and are a necessary step towards a complete understanding of how a retina of the right size and cell composition is generated from a set of equipotent progenitors.

### Plexiform layer formation

SoFa lines are potentially attractive model systems in which to study how synaptic specificity and connectivity are achieved in the developing retinal neuropils – the outer (supplementary material Fig. S2A-D) and inner (supplementary material Fig. S2E-N) plexiform layers. By examining the plexiform layers over time or along the wave front of differentiation, it should be possible to develop a better framework for understanding how these neuropils are constructed. For example, supplementary material Movie 5 shows that BCs (blue), although born late, send their processes into the inner plexiform layer early, consistent with our previous findings using a pairwise experimental analysis (Randlett et al., 2013). In addition, supplementary material Fig. S2E-H shows that at 76 hpf, the axonal processes of BCs already display a simple but discrete sublamination pattern in the inner plexiform layer, whereas the processes of ACs and RGCs seem less patterned.

An intriguing issue for developmental neurobiologists is whether cells that are closely related by lineage have a greater preference to connect to each other than cells that are less closely related. Indeed, in the visual cortex of mice, there are indications that clonally related cells may preferentially share orientation tuning and thus connect to each other (Li et al., 2012; Ohtsuki et al., 2012). In the SoFa2 clones (Fig. 4K and supplementary material Movie 4), the proximity of cellular processes within the outer and inner plexiform layers of these clonally related neurons is suggestive of functional connectivity. The expression of a cytoplasmic calcium indicator in retinas where there is labelling of single-cell derived clones could be a very useful way to accurately measure the relative probabilities of clonal versus non-clonal functional connectivity. When cell type identity is also known, as in Spectrum of Fate retinas and clones, one can begin to piece together a more precise understanding of how the circuitry of the retina develops.

### Fate switching and the relationship between fate and proliferation

**Fig. 5.**
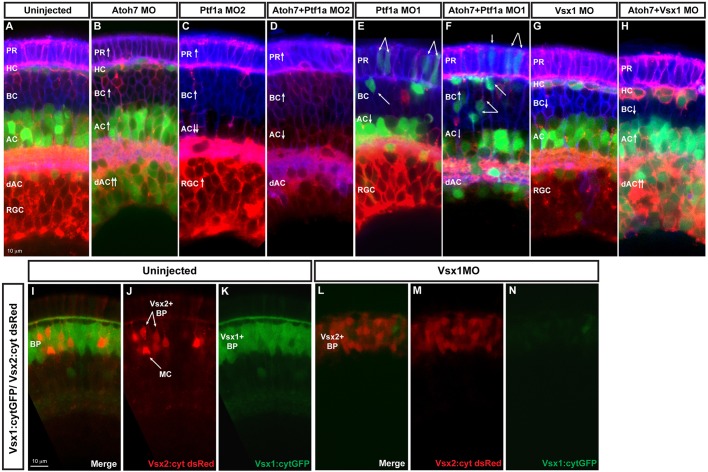
**Fate switching.** Images show examples of loss-of-function analysis performed using SoFa. (A) Uninjected SoFa1 retina. (B) Atoh7 morphant retina in which there is a decrease in RGCs and a clear increase in the number of dACs and ACs (red and green cells), BCs (blue cells) and PRs (purple cells). (C,E) Ptf1a MO1 and MO2 morphant retinas. Ptf1a MO2 (C) is a more potent translation-blocking morpholino that interferes with both Ptf1a and Ptf1a:cytGFP expression. Ptf1a MO1 (E) is a splice-blocking morpholino, which consequently knocks down the translation of Ptf1a, but not ptf1a:cytGFP, so cells that would have been ACs and HCs can be seen in their transfated states. In these morphants, we see reductions in the generation of ACs, dACs and HCs, and an increase in the number of RGCs (red), BCs (blue) and PRs (purple). (D,F) Atoh7/Ptf1a double morphants, showing a retina devoid of RGCs and HCs, with a significant reduction in the number of dAC and ACs (red only), a significant increase in the number of PRs (purple) and BCs (blue), and an increased amount of transfating. (G,H) Knockdown of Vsx1 alone leads to a small decrease in BCs because of compensation from Vsx2, whereas simultaneous knockdown of Vsx1 and Atoh7 leads to increases in ACs [especially displaced ACs (red and green) and PRs (purple)]. (I-K) Cross sections of uninjected Vsx1:cytGFP; Vsx2:cyt dsRed retinas. Most BCs are green, indicating that they are Vsx1+. (L-N) Knockdown of Vsx1 results in loss of most Vsx1+ BCs, but there is almost a complete compensation in BC numbers via an increase in Vsx2+ BCs.

The genes whose promoters drive the expression of the FPs in the SoFa lines are themselves required for the genesis of distinct neuronal types in the retina. Thus, for example, *atoh7* mutants and morphants lack RGCs, whereas *ptf1a* mutants and morphants are deficient in the generation of ACs and HCs (Kay et al., 2001; Jusuf et al., 2011; He et al., 2012; Randlett et al., 2013). In agreement with previous results, analysis of these morphant retinas suggests that instead of just losing these cell types from the retina, there are compensatory mechanisms by which cells, rather than die, appear to switch their fates. For example, when SoFa fish are injected with *atoh7* morpholino, there is a loss of RGCs but a dramatic increase in cytGFP-expressing cells in the RGC layer, which corresponds to an increase in ACs (Fig. 5B). Reciprocally, in fish injected with the translation-blocking morpholino to *pft1a* (MO2), there is a loss of ACs and HCs but a large increase in gapRFP-expressing RGCs (Fig. 5C). These changes are evident when one compares the thickness of the green or red layers. In both *atoh7* and *ptf1a* single morphants, there are also increases in the number of PRs and BCs, and such an increase becomes more prominent in *atoh7*/*ptf1a* double morphants, which completely lack RGCs and HCs, and have a severe reduction in the number of ACs (Fig. 5D). Fate switching can be tracked in *ptf1a*-expressing cells if the Ptf1a transcription factor is knocked down using a splice morpholino (MO1) that does not affect the expression of ptf1a:GFP (Fig. 5E). It should be noted, however, that MO1 does not cause as effective a phenotype as MO2, which blocks both Ptf1a and ptf1a:GFP expression. Nevertheless, using the splice-blocking morpholino, we see cells with BC and PR morphologies arising from cells expressing *pft1a:GFP* (Fig. 5E). When, in addition to Ptf1a, we knock down Atoh7 to interfere with RGC development, the resulting retinas are composed of an even higher proportion of BPs that have switched fates (Fig. 5F).

Fate switching in *atoh7* and *ptf1a* mutant and morphant fish has been noted previously (Kay et al., 2001; Jusuf et al., 2011; He et al., 2012), and it is not difficult to imagine that if cells are prevented from taking early fates, they may still be able to take later fates. But what happens if one prevents later fates, such as BC fate? In zebrafish, are two types of BCs: Vsx1+, which comprise 17 out of the 18 types of BCs; and Vsx2+, the single remaining type (Vitorino et al., 2009). We therefore knocked down Vsx1 with a translation-blocking morpholino. The result is a dramatic decrease in the number of Vsx1+ BCs, which is accompanied by a significant increase in the number of Vsx2+ BCs (Fig. 5I-N). Although there is not an obvious increase in RGCs or ACs (Fig. 5G), simultaneous injection of Vsx1 and Atoh7 morpholinos result in retinas with a huge increase in ACs, most of which are displaced, as well as increased numbers of PRs (Fig. 5H). This unique and almost complete set of single knockdowns clearly shows that fate re-specification is possible during early retina development, while retaining the overall topography of the retina. The Spectrum of Fates approach thus opens up powerful opportunities to investigate how different transcription factors, or other molecules that are thought to affect fate choice (working alone or in combination), affect relative fate potentials, thus revealing possible hierarchies of fate choice within a stochastic framework.

### Dissociated SoFa

**Fig. 6.**
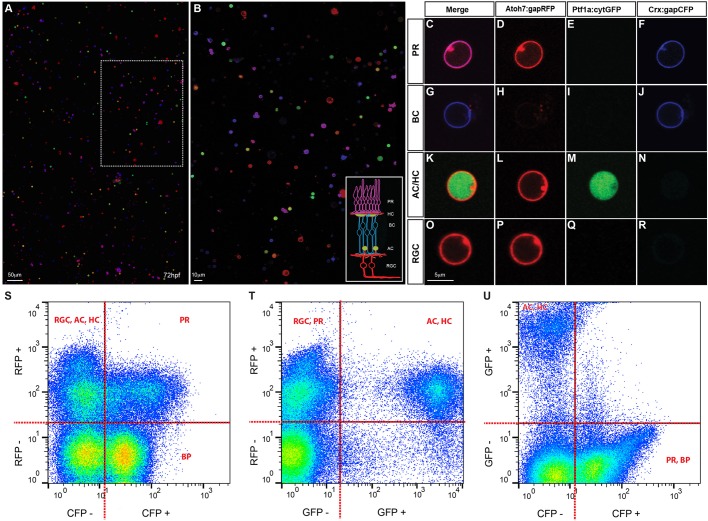
**Cell type identification in dissociated retinal cultures.** (A,B) SoFa1-dissociated retinal cells and correspondent high magnification inset, showing that all cell types can be easily identified by their specific combination of colours. (C-R) Representative images of cells of each cell type (red, green and blue channels shown separately and merged). PRs are labelled in purple, as they express both Atoh7 and Crx (C); BCs are labelled in blue, as most of them express exclusively Crx (G); AC and HC are labelled in red and green, as they express both Atoh7 and Ptf1a (K); and RGCs are labelled in red, as all express Atoh7 (O). (S-U) Flow cytometry profiles of dissociated SoFa1 retinas, showing that it is possible to sort the different retinal populations. (S) RFP and CFP profile; (T) RFP and GFP profile; and (U) GFP and CFP profile.

Although the SoFa lines were designed primarily to follow developmental processes *in vivo*, many questions in cell biology are most directly approached using primary cells in culture. In a tissue that is composed of many cell types, specific antibodies are usually necessary to identify these cell types when they are dissociated *in vitro*; and if one wants to study the cells live, such antibodies must recognise cell surface antigens and yet not interfere with cell function. When SoFa retinas are dissociated, however, neuronal types are immediately identifiable independently of their morphology, by virtue of their fluorescent spectra (Fig. 6A-R). These cells can be counted, sorted by flow cytometry (Fig. 6S-U), and subsequently used for transcriptomic and proteomic studies. They can also be cultured at high or low density, and in specific combinations, to study the intrinsic physiological properties of these cells or to investigate how cells of one type interact with cells of other types in a range of developmental assays.

## DISCUSSION

We describe a Spectrum of Fates approach for the study of zebrafish retinal development, allowing real time imaging of all types of retinal neurons simultaneously. In SoFa lines, as each major cell type fluoresces with a distinct spectral colour, we can examine the consequences of genetic and pharmacological manipulations, and provide an immediate read-out of a variety of cell type-specific behaviours *in vivo* or *in vitro* without the need for immunohistochemical procedures.

We believe that SoFa lines can be powerful tools with which to understand the mechanisms of cell fate acquisition. By adopting a Spectrum of Fates approach to look at cell diversification in morphants and/or mutants defective in transcription factors, signalling systems, cell cycle components and other pathways, both at clonal and populational level, we should gain insights into the hierarchies of fate choice during retinal development. In this paper, we have shown that in morphants for *atoh7* and *ptf1a*, retinal progenitors are prevented from taking early fates such as RGCs and ACs, whereas *vsx1* morphants have a dramatic decrease in Vsx1+ BCs. In each case, there is a compensatory increase in other cell types, which is even more dramatic when we prevent two or more fates simultaneously, although the overall topography of the retina is maintained. Further quantitative analysis of fate switching in different knockdowns will certainly contribute to establishing the rules governing the relationships between the cellular fates.

Spectrum of Fates is complementary to Brainbow technology: while the stochastic combination of FPs of Brainbow provides a way to distinguish all adjacent cells, SoFa lines can be used to assign fates to all cells. Hence, by using a combination of the Zebrabow (used to label all progenitors and their clonal descendants by cytoplasmic FPs) and SoFa2 (which specifically labels the membranes of each cell type in distinct colours), we should be able in principle to track all clones by their cytoplasmic spectrum and all the cell fates in these clones independently by their membrane spectra. Adding yet other orthogonal markers, such a calcium indicators, should make it possible to see the ontogeny of synaptic information transfer across the specific cells in the developing circuitry of the retina.

In addition to clonal analysis in the developing retina, the SoFa lines should be useful to study regeneration in the adult retina. Following injury to the central retina it will be possible to characterize the origin, the composition and eventually the dynamics of multicolor clones arising in the wounded region.

In this study, we showed that SoFa lines can be especially useful for determining the respective order of differentiation events between interacting cells. An interesting example is the wave of differentiation in the zebrafish retina, which sweeps across the retina and expands towards the periphery. In SoFa fish lines, this wave is beautifully apparent in all cell types and one can see and quantify the relationships of cells of different types that are differentiating. We found that there is a high Index of Interdigitation between cell types at early stages of differentiation, and these indexes then reach a minimum before HCs migrate apically and dACs migrate basally. This scenario is distinct from what has been proposed – that there are several waves of final cell division and differentiation, so that the retina is built layer by layer (Livesey and Cepko, 2001). Our results are more in line with the variability in patterns of clonal histogenesis seen in the mouse, frog and zebrafish retinas (Holt et al., 1988; Belecky-Adams et al., 1996; Rapaport et al., 2004; Vitorino et al., 2009; He et al., 2012). The stochastic mechanisms operating within clones lead to the near simultaneous birth of several cell types within very small regions. This is the likely cause of the high level of early interdigitation, and suggests that cells of different types may use each other to find their correct lamina positions.

Brainbow produces visual striking images that highlight the intricacy of neural networks. However, the biological significance of these images is not always immediately apparent to the observer, as the cellular colours are generated through a stochastic mechanism. With Spectrum of Fates, although not yet as delicately nuanced in terms of spectral combinations, we can immediately appreciate the developing cellular architecture within a tissue. In the future, it will be important to introduce more colour combinations into the SoFa lines, so that Müller cells, the intrinsic retinal glial type, are visible, and the two inhibitory retinal neuron types, ACs and HCs, can firmly be distinguished from each other. Eventually, it should be possible to distinguish various subtypes of the different major classes of cells. There is a visually attractive appeal to the present and hopefully future SoFa lines. Yet here, in addition to these aesthetic issues, we hope we have successfully highlighted the possibilities of using a Spectrum of Fates approach to answer questions of cell fate and cell differentiation in the retina.

## MATERIALS AND METHODS

### Animals and transgenic lines

Zebrafish lines were maintained and bred at 26.5°C. Embryos were raised at 28.5°C and staged in hours post fertilisation (hpf). Embryos were treated with 0.003% phenylthiourea (PTU, Sigma) at 8 hpf to delay pigmentation, and were anaesthetised by 0.04% MS-222 (Sigma) prior to live imaging or fixation. Two-month old fish were euthanised by anaesthetic overdose in MS-222. All animal work was approved by Local Ethical Review Committee at the University of Cambridge and performed according to the protocols of UK Home Office licence PPL 80/2198.

Atoh7:gapRFP (Zolessi et al., 2006), Ptf1a:cytGFP (Godinho et al., 2005), Ptf1a:Gal4/UAS:gapYFP (Pisharath and Parsons, 2009; Williams et al., 2010), Vsx1:cytGFP and Vsx2:cyt dsRed (Kimura et al., 2006; Vitorino et al., 2009) transgenic lines have been described previously. The Crx:gapCFP transgenic line was generated by injection of the Crx promoter driving gap CFP DNA construct (Suzuki et al., 2013). The polychrome transgenic lines Atoh7:gapRFP/Ptf1a:cytGFP/Crx:gapCFP (SoFa1) and Atoh7:gapRFP/Ptf1a:Gal4/UAS:gapYFP/Crx:gapCFP (SoFa2) were generated by crossing single with double transgenic lines.

### Transplantation

SoFa1 or SoFa2 transgenic embryos (donors) and wild-type embryos (hosts), were dechorionated with pronase (0.6 mg/ml) at the blastula stage (4 hpf) and positioned in a transplantation mould (Adaptive Science Tools). Fewer than five donor cells were transplanted into the animal pole of host cell mass, where the cells are expected to develop into retina cells (Kimmel et al., 1990). Donor and host embryos were kept at 28.5°C for 2 hours on the transplantation mould, then transferred to agarose-coated dishes and then returned to 28.5°C. For live imaging, hosts were screened at 40 hpf on an upright fluorescent microscope to select those where Atoh7 expression has just started. For fixed samples, hosts were screened at 48 hpf to select those that express all three FPs in small retinal clones.

### Confocal image acquisition and analysis

Live embryos at desired developmental stages were collected and embedded in 1% low melting agarose with the proper orientation. Two-month-old retinas were collected from euthanised fish, fixed in 4% PFA and processed as described previously (Thummel et al., 2008). The eyes were sectioned at 50 μm. Cryosections were dried for 1 h at room temperature and mounted using Fluorsave (Calbiochem). Retinal clones or entire retina were imaged under 60× (NA=1.30) or 30× (NA=1.05) silicon oil objectives on the inverted laser-scanning confocal microscope (Olympus FV1000).

All the images were acquired by comparable settings (1024×1024 or 1600×1600 resolution, 12.5 μs/pixel scanning speed, 1-2 μm optical section). Image analysis was performed using ImageJ or Volocity Software (Perkin Elmer).

### *In vivo* live imaging

Dechorionated embryos were collected at desired time points, such as 32 hpf (entire retina imaging) or 40 hpf (retinal clones). After screening, embryos were embedded in 1% low melting agarose in a custom-made imaging dish, in imaging medium (1× Steinberg, 0.02% MS222 and 0.003% PTU). Four-dimensional live imaging was conducted on the inverted laser scanning confocal microscope (Olympus FV1000) at 28.5°C controlled by a customer-made heating block. Optical sections at 1-2 μm separation were taken to cover the region of the retina containing the cells of interest. Four-dimensional movies were analysed using Volocity Software.

### Interdigitation analysis

To calculate the Index of Interdigitation in single images of the retinal wave, we first define a polar coordinate system by tracing the apical surface and then fitting a circle, with centre c, defining the origin. We then trace the maximal apical/minimum basal radial position, t(θ), of the cell layer of interest as a function of azimuth, and define the Index of Interdigitation, I(θ,Δθ), as the standard deviation of t(θ) over each θ±Δθ segment. In our analysis, we choose Δθ=10°, which results in sufficient samples to extract a meaningful standard deviation of t(θ) while being small enough to observe changes across the retina. The method of calculating the Index of Interdigitation in movies is similar to that of in single images, except that we define a polar coordinate system by tracing the apical surface and fitting a circle in every frame, and the origin is defined as the average centre of the fitted circles. The Interdigitation analysis was performed using MATLAB (MathWorks).

### Morpholino injection

Antisense translation blocking morpholinos were obtained from Gene Tools, reconstituted as 1 or 3 mM stock solutions in water, and injected into the yolk of one- to two-cell stage embryos. As previously described, injection of 10-12 ng of Ptf1a-MO2 (5′-TTGCCCAG-TAACAACAATCGCCTAC-3′) resulted in a retina devoid of HCs and most ACs and dACs, whereas injection of 12 ng of Ptf1a-MO1 (5′-CCAACACAGTGTCCATTTTTTGTGC-3) resulted in a retina with a significant reduction on HCs and a moderate reduction on ACs and dACs (Jusuf et al., 2011). Injection of 2 ng of Atoh7 MO [5′-TTCATGGCTCTTCAAAAAAGTCTCC-3′ (Pittman et al., 2008)] resulted in a retina devoid of RGCs, phenocopying the zebrafish *lakritz*/Atoh7 mutant phenotype (Kay et al., 2001). Injection of 2 ng of Vsx1MO (5′-AATTCACTTTCTCTCTCAGACTGGT-3′) resulted in a retina with significant reduction of Vsx1+ BCs and concomitant increase of Vsx2+ BCs. At 72 hpf, morpholino-injected SoFa1 embryos were fixed in 4% PFA and imaged as described above.

### Cell dissociation and flow cytometry

Cell suspensions were prepared from freshly dissected retinal tissue. SoFa1 embryos (72 hpf) were transferred to cold Ca^2+^-free medium [116.6 mM NaCl, 0.67 mM KCl, 4.62 mM Tris; 0.4 mM EDTA (pH 7.8) (Harris and Messersmith, 1992)] supplemented with 100 µg/ml of heparin and 0.04% MS-222. Fifteen to 20 eyes were transferred to glass Petri dishes. Without disturbing the retinas, the Ca^2+^-free medium was removed and 0.25% Trypsin-EDTA was added. Following a 10 min incubation at 37°C, the trypsin was removed, and the retinas were mechanically dissociated by pipetting using flame-pulled glass Pasteur pipettes. For confocal imaging, single cell suspensions were plated into 35 mm imaging dishes, seeded for 1 h at 28.5°C and imaged. For flow cytometry, single cell suspensions were analysed using a Fortessa flow cytometer and analysed using FlowJo (Tree Star). Retinal cells were discriminated based on the forward (FSC-A) and side-scatter (SSC-A) profiles. Single-colour RFP, CFP and GFP cells were used to generate a compensation matrix.

## Supplementary Material

Supplementary Material
