# Supplementary material for: Spectrum of Fates: a new approach to the study of the developing zebrafish retina
Source: Development. 2014 May;141(9):1971–80. doi: 10.1242/dev.104760 (PMC3994774; doi:10.1242/dev.104760)
Supplement: Supplementary Material [file supp_141_9_1971__index.html]

Spectrum of Fates: a new approach to the study of the developing zebrafish retina — Supplementary Material 

# Spectrum of Fates: a new approach to the study of the developing zebrafish retina

## DEV104760 Supplementary Material

**Files in this Data Supplement:**

- **Supplementary Material**
