# Supplementary material for: Spectrum of Fates: a new approach to the study of the developing zebrafish retina
Source: Development. 2014 May;141(9):1971–80. doi: 10.1242/dev.104760 (PMC3994774; doi:10.1242/dev.104760)
Supplement: Supplementary Material [file supp_141.9.1971_DEV104760.pdf]

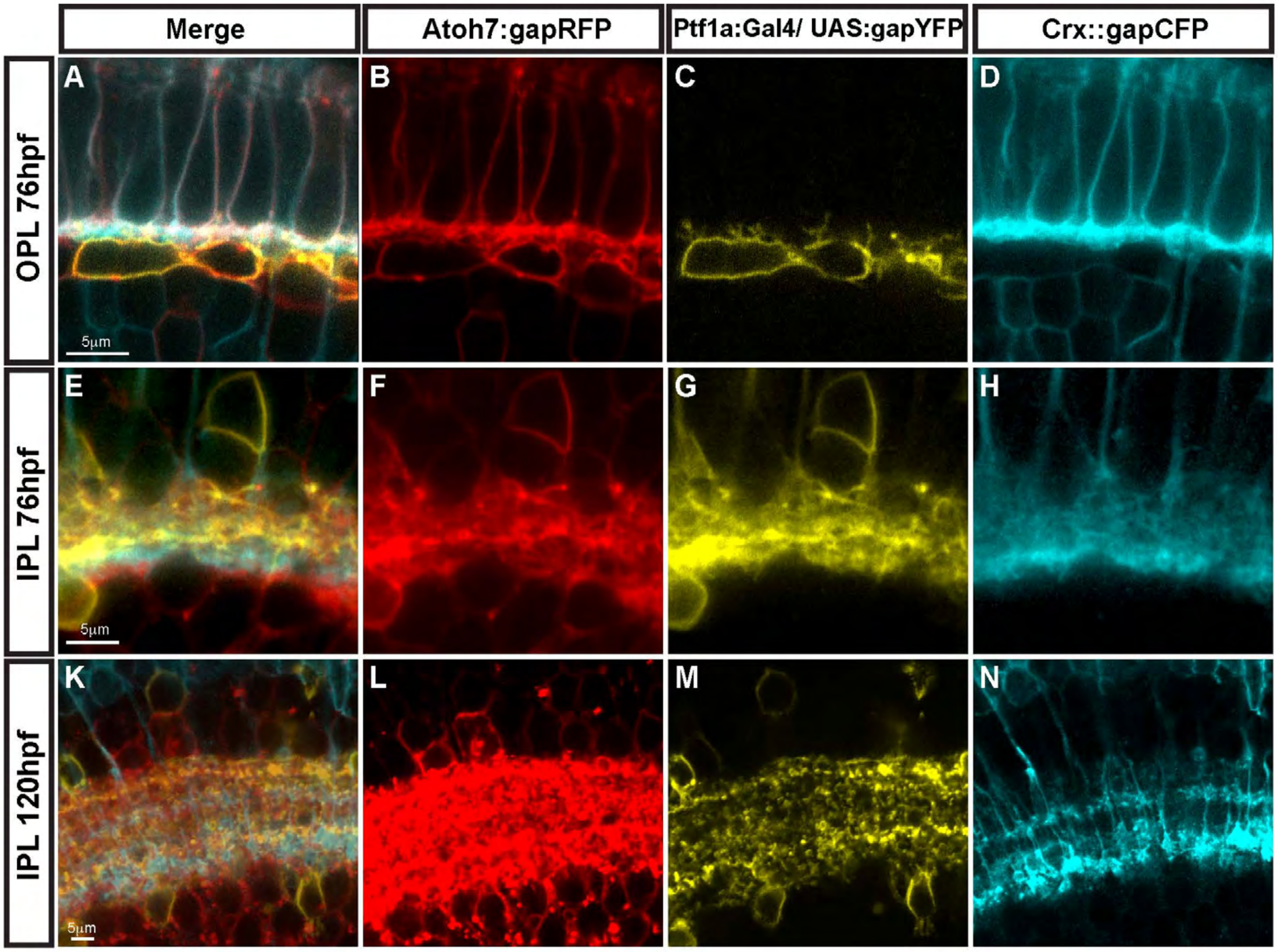

Supplementary Figure 1. Dynamics of the retina differentiation wave
Time lapse series of a developing SoFa1 retina, showing the RGC, AC and PR differentiation waves (A-L). The figure demonstrates that although some cell types tend to differentiate before others, there is a substantial overlap in the generation of the different fates.

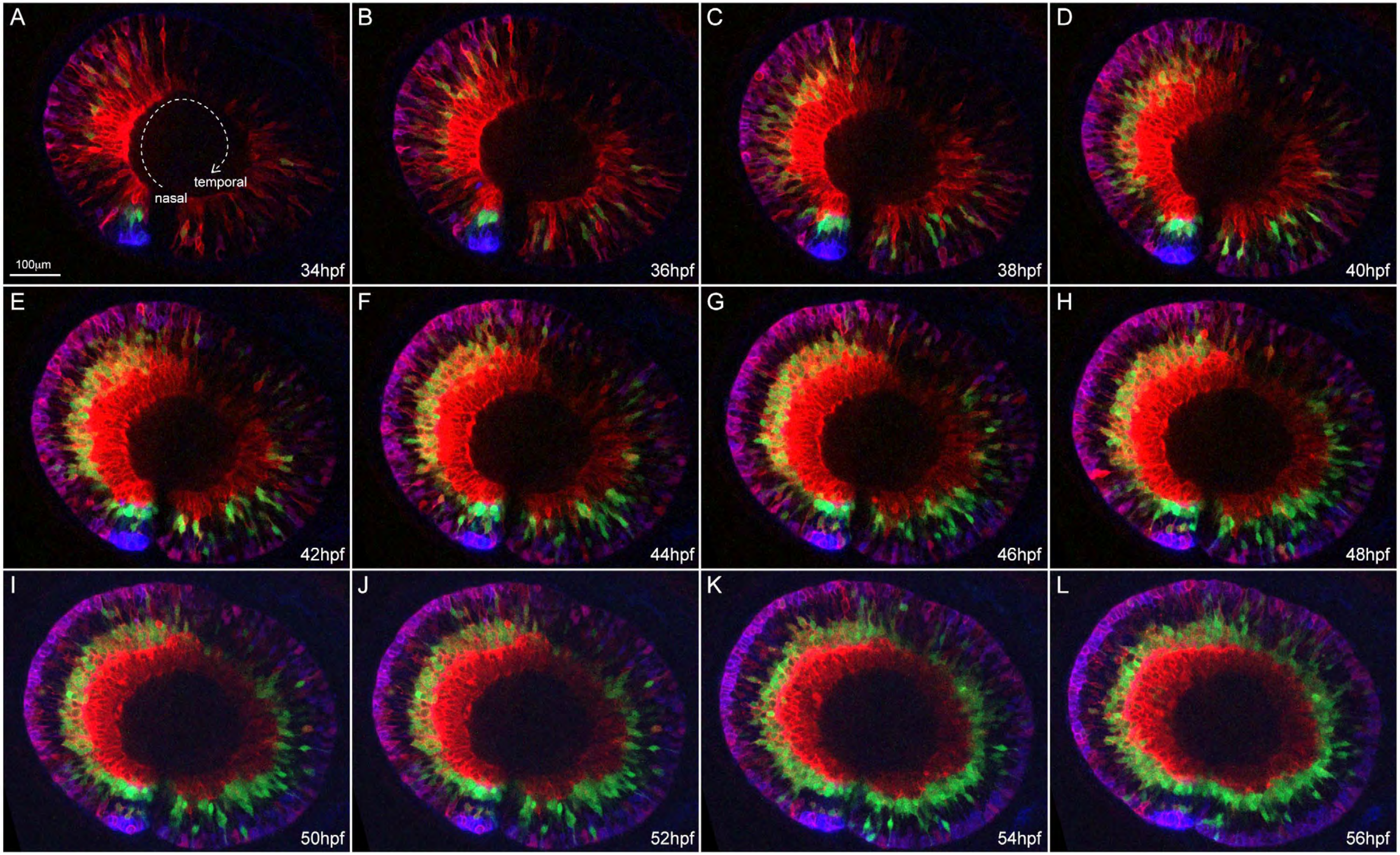

Supplementary Figure 2. SoFa2 Plexiform Layers
High magnification views of the outer (OPL) and inner (INL) plexiform layers of SoFa2 retinas, showing that by labeling all retinal neurons with a membrane tagged FP it is possible to study the process of lamination of the plexiform layers (compare E with K) and potentially connectivity in the retina. (A-D) 76hpf OPL. (E-H) 76hpf INL. (I-L) 120hpf INL.

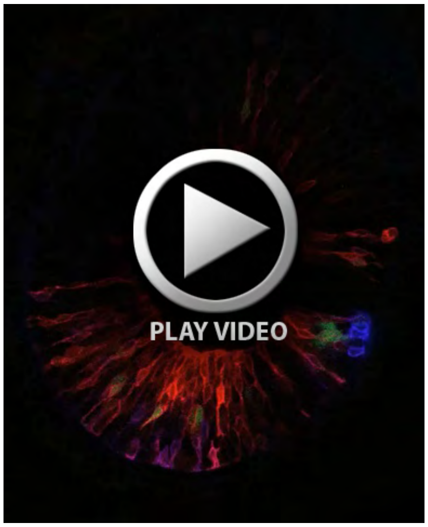

Supplementary Movie 1:
Time lapse showing the retina differentiation waves (in Fig.S1), starting from 34hpf to 56hpf. Images were acquired every hour.

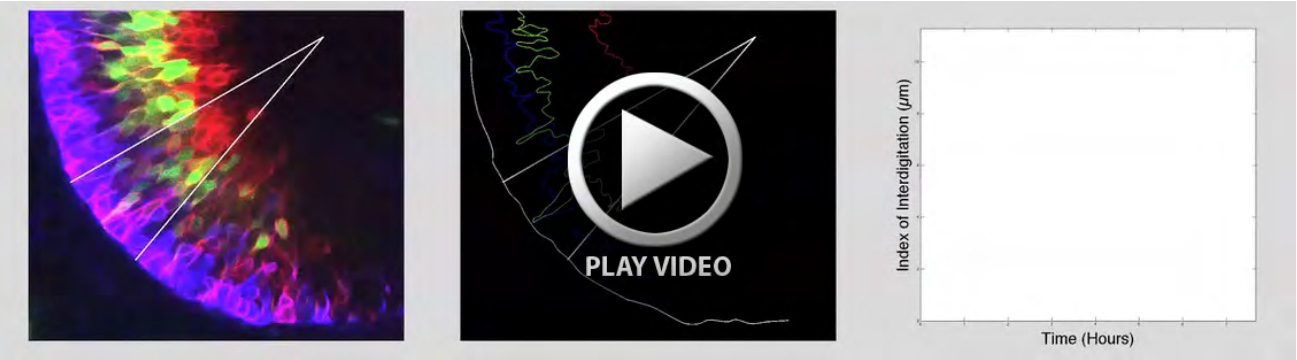

Supplementary Movie 2:
(A) Time lapse of a SoFa1 retina showing the interdigitation of the cell types during retinal lamination, starting at 48hpf. Single plane, images acquired every 20min. (B) Traces of the apical surface (white), the basal side of the PR layer (blue), the apical side of the IN layer (green) and apical side of the RGC layer (red) from when they first appear continuous across time (C) Graph showing how the Index of Interdigitation varies across time for the PR layer (blue), the apical side of the IN layer (green) and the apical side of the RGC layer (red).

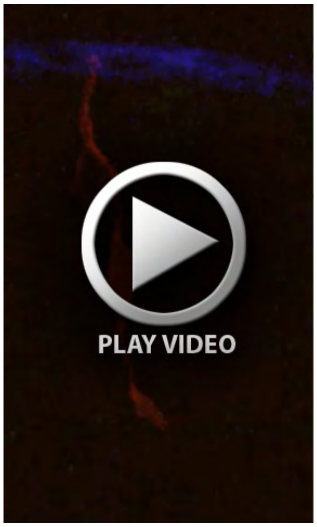

Supplementary Movie 3:
Time lapse of a multicolor SoFa1 clone (in Fig. 4A-I) developed from 45hpf to 55hpf, showing the Atoh7, Crx and Ptf1a transgenes turning on, as well as the migration of the multicolor cells towards their appropriate location within the retina.
3D projection, images acquired every hour.

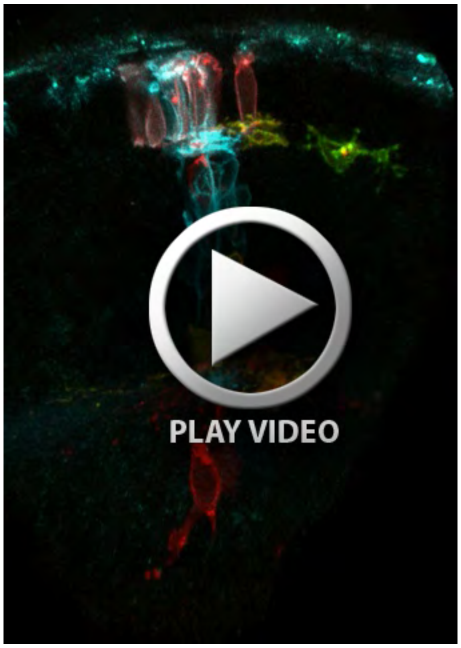

Supplementary Movie 4:
Rotation of SoFa2 clone from Figure 4K.

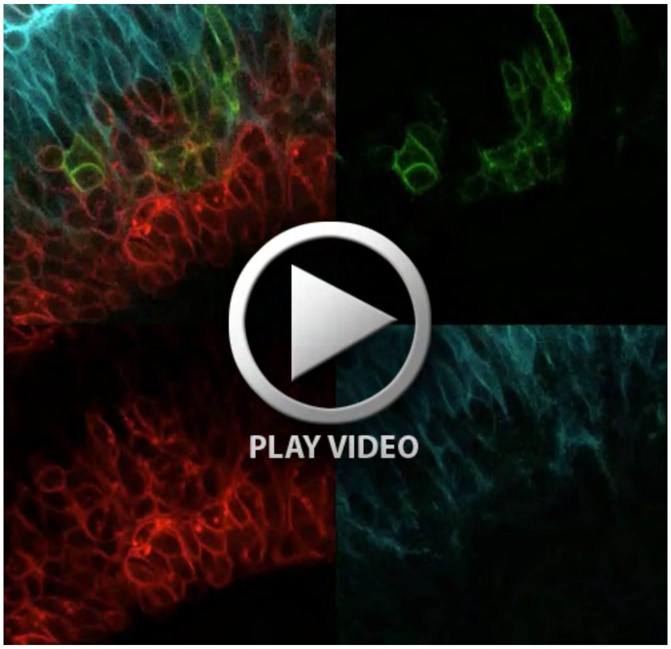

Supplementary Movie 5:
Time lapse of a SoFa2 retina showing the formation of the inner plexiform layer.
3D projection, images acquired every 20min.
